# Disrupted structural covariance network in presbycusis patients: evidence from a large sample mri-based morphometric study

**DOI:** 10.3389/fnagi.2026.1889317

**Published:** 2026-07-24

**Authors:** Qi Yan, Ying Wang, Li Xu, Baoxu Liu, Bin Zhu, Song’an Shang, Bing Guan

**Affiliations:** 1 Department of Otolaryngology, Northern Jiangsu People’s Hospital, Institute of Translational Medicine, Medical College, Yangzhou University, Yangzhou, China; 2 Department of Radiology, Northern Jiangsu People’s Hospital, Institute of Translational Medicine, Medical College, Yangzhou University, Yangzhou, China

**Keywords:** brain functional network, cognitive, f-MRI, presbycusis, structural covariance network

## Abstract

**Background:**

Presbycusis is characterized by progressive age-related hearing loss and is often associated with cognitive decline and mental health disorders.

**Objective:**

This study aimed to investigate gray matter volume (GMV) alterations and structural covariance network (SCN) reorganization in presbycusis patients via magnetic resonance imaging (MRI) and graph theory analysis.

**Methods:**

A total of 129 presbycusis patients and 121 age-matched healthy controls (HCs) underwent neuropsychological assessments and high-resolution T1-weighted MRI. The voxel-based morphometry (VBM) method at the region of interest (ROI) level and the graph theory analysis method of the SCN based on GMV were used to study the impact of presbycusis on brain structure.

**Results:**

Patients presented altered nodal topological properties in multiple brain regions, including the right insula, left hippocampus, bilateral superior frontal gyrus, and occipital cortices. Significant differences were observed in the characteristic path length (Lp) and normalized characteristic path length (λ) between the groups (*p* < 0.05). Cognitive assessments revealed impairments in executive function, memory, and visual-spatial abilities (*p* < 0.05).

**Conclusion:**

Presbycusis is associated with GMV reduction and SCN reorganization across sensory, cognitive, and emotional brain networks. Compensatory neural mechanisms, such as enhanced visual processing, may alleviate the adverse impacts of auditory deprivation. These findings elucidate the neuroanatomical basis of cognitive decline in presbycusis and highlight the necessity of early clinical intervention.

## Introduction

Presbycusis, the most prevalent age-related hearing disorder, affects more than 30% of individuals aged 65 and older, ranking third among the most common geriatric chronic conditions after hypertension and arthritis (GBD 2015 Disease and Injury Incidence and Prevalence Collaborators, 2016). Presbycusis is not only a result of aging but also a disease that leads to hearing loss with age, characterized by an increased number of congenital cochlear nucleus neurons (Hinojosa and Nelson, 2011; Rumschlag et al., 2021). Presbycusis can lead to central nervous system processing disorders, resulting in poor discrimination and localization abilities in noisy environments (Ren et al., 2021; Parham et al., 2011), beyond auditory deficits, it is linked to cognitive impairment, depression, and social isolation (Xing et al., 2020; Li et al., 2023). At the cortical level, hearing loss disturbs neural afferent inputs to the primary auditory cortex and receptive language regions, further impairing higher-order language association areas that mediate semantic processing and sound interpretation (Ren et al., 2018). Cumulative clinical and neuroanatomical evidence indicated that presbycusis involves not only peripheral cochlear degeneration but also large-scale brain network remodeling, which critically contributes to disease progression.

According to the Lancet Commission report on dementia prevention, intervention, and care, hearing loss is ranked among the leading modifiable risk factors for dementia (Livingston et al., 2024). Alterations in brain structure and function have been hypothesized to serve as the core causal mechanisms underlying the link between hearing loss and dementia (Whitson et al., 2018). A growing body of neuroimaging studies has explored the correlations between age-related hearing loss and brain structural and functional biomarkers, with existing reviews systematically summarizing relevant evidence, particularly functional MRI findings (Jafari et al., 2021). Consistent findings from clinical investigations and large-scale population cohorts demonstrate that hearing deficits correlate with adverse neuroimaging phenotypes, including cortical atrophy, reduced gray matter volume, and aberrant functional connectivity.

Despite substantial progress in characterizing functional network abnormalities in presbycusis, prior studies have largely focused on functional connectivity rather than structural brain architecture. Given that brain structure fundamentally constrains neural function, the whole brain operates as an integrated structural and functional network for information transmission. Therefore, investigating structural brain patterns is indispensable for comprehensively understanding presbycusis-related neural remodeling. Magnetic resonance imaging (MRI), a pivotal tool in neuroimaging research, enables sensitive tracking of changes spanning from microstructural degradation to morphological atrophy. Structural covariance network (SCN), which reflect synchronized gray matter variations across brain regions, provide a novel framework to explore large-scale neural reorganization in presbycusis. Enabled by automated whole-brain analytical approaches for large-scale *in vivo* datasets, SCN analysis delineates macroscopic structural network architectures built upon anatomical connectivity and cross-regional functional integration (Spreng and Turner, 2013). Graph theoretical metrics derived from SCN data further reveal global topological organization of brain morphological covariation, as well as the spatial distribution of functional hubs that exhibit intensified structural coupling with distributed brain regions (Yun et al., 2020).

SCNs serve as a complementary approach to other connectivity analyses and have emerged as a critical tools for exploring brain organization. Given the pivotal role of neural remodeling in hearing loss and the unique advantages of SCNs, this present study integrated morphological data and graph theory to compare SCN patterns between presbycusis patients and healthy controls (HCs). Our objectives are twofold: (1) to identify neuroanatomical MRI markers specific to presbycusis by analyzing gray matter covariance; and (2) to characterize alterations in topological properties (e.g., small-worldness, path length) of SCN in presbycusis. Overall, this study leverages SCN topological analysis to explore MRI-derived neuroimaging biomarkers and elucidate the potential neural mechanisms linking age-related hearing loss, brain structural remodeling, and cognitive impairment.

## Materials and methods

### Participants

Elderly participants were consecutively recruited from the Department of Otolaryngology and Department of Geriatrics of Northern Jiangsu People’s Hospital from August 2022 to December 2024, including both inpatients and outpatients. A frequency matching strategy was applied in this study via SPSS software. Participants in the presbycusis group were stratified by sex, age bracket, and educational level. Healthy controls (HCs) with normal hearing were randomly selected from a pool of healthy volunteers to ensure consistent proportional distributions of the three stratified variables between the two groups. After rigorous eligibility screening, a total of 129 participants who met the diagnostic criteria for presbycusis were included in the patient group, and 121 age-, sex-, and education-matched individuals with normal hearing were enrolled as controls (mean age: 63.83 ± 3.46 years). This study was conducted in strict adherence to the principles of the Declaration of Helsinki. Ethical approval and participant informed consent were obtained prior to enrollment. All participants consented to the publication of relevant research data. Due to ethical privacy protection regulations, the raw data generated in this study are not publicly available.

### Human ethics declaration

This study was conducted in strict accordance with the principles of the Declaration of Helsinki. The research protocol was reviewed and approved by the Ethics Committee of Northern Jiangsu People’s Hospital (Approval No. 2025js65). All procedures involving human participants were carefully evaluated to minimize potential risks and maximize benefits. All patients signed the informed consent form. All data were fully anonymized and de-identified prior to analysis, and no potential risks to participants’ rights and welfare were involved. The entire study procedure complied with local regulatory requirements and standardized ethical guidelines for human subject research. Raw individual data are not publicly available due to ethical privacy restrictions.

### Inclusion criteria

#### Presbycusis group

Participants were included if they were aged ≥ 60 years, right-handed, with a minimum of 6 years of formal education, and presenting with progressive bilateral sensorineural hearing loss lasting more than 3 months. Pure-tone audiometry was performed to evaluate hearing thresholds at 0.25, 0.5, 1, 2, 4, and 8 kHz, and participants were included if their four-frequency pure-tone average (500, 1,000, 2,000, and 4,000 Hz) exceeded 25 dB HL. All audiometric assessments were completed in a sound-attenuated examination room with controlled ambient lighting to enable real-time participant observation and eliminate visual cue interference. Participants were seated in a fixed posture and instructed to turn off personal electronic devices and remove accessories that might interfere with transducer placement. Testing was implemented using a clinically calibrated audiometer (Otosuite) complying with IEC 60645 standards. Air conduction thresholds (125–8,000 Hz) were measured via TDH-39 supra-aural headphones, while bone conduction thresholds (250–4,000 Hz) were acquired using a B71 bone vibrator, with responses recorded through a handheld patient response button. Ambient noise levels were continuously monitored via fast Fourier transform (FFT) analysis to ensure the noise floor remained within standard permissible limits throughout testing. Audiometric threshold determination strictly followed the Hughson–Westlake protocol: otoscopic examination was first performed to exclude external auditory canal occlusion; transducers were accurately positioned over the ear canal for air conduction and the mastoid process for bone conduction; testing started at 1,000 Hz, with stimulus intensity decreased in 10 dB steps and increased in 5 dB steps. Hearing threshold was defined as the lowest intensity yielding a response reliability of no less than 50%. Contralateral masking was applied when interaural attenuation exceeded 40 dB.

#### Control group

Healthy individuals with normal hearing thresholds ( ≤ 25 dB HL) across all tested frequencies (0.25–8 kHz), matched for age, sex, and educational level with the presbycusis cohort.

### Exclusion criteria

Individuals with any of the following conditions were excluded: (1) auditory disorders, including sudden hearing loss, conductive hearing loss, noise-induced hearing loss, Meniere’s disease, chronic otitis media, ear surgery history, and long-term use of ototoxic drugs or hearing aids; (2) neurological or psychiatric disorders, including stroke, traumatic brain injury, Parkinson’s disease, Alzheimer’s disease, epilepsy, severe depression, and other cognitive-impairing diseases; (3) severe systemic diseases, including anemia, thyroid dysfunction, malignant tumors, MRI contraindications, and severe visual or gustatory dysfunction; (4) unhealthy lifestyle factors, including heavy smoking ( > 5 cigarettes per day) and chronic alcoholism.

### Group matching procedure

To reduce selection bias and guarantee homogeneous baseline characteristics between the two groups, PSM was implemented for demographic standardization. Presbycusis patients and healthy controls were matched at a 1:1 ratio using a nearest-neighbor matching algorithm with a caliper of 0.05. Age, sex, and years of education were defined as core matching covariates. This standardized matching procedure ensured that demographic confounders were well controlled, thereby improving the reliability and validity of subsequent structural covariance network analyses.

### Pure-tone audiometry assessment

All audiometric examinations were conducted in a sound-attenuated booth that complied with ISO background noise standards. A clinically calibrated audiometer (Otosuite) meeting IEC 60645 specifications was used for testing. Air-conduction thresholds were measured at octave frequencies ranging from 250 to 8,000 Hz using TDH-39 supra-aural headphones, while bone-conduction thresholds (250–4,000 Hz) were recorded via a B71 bone vibrator. Ambient noise levels were continuously monitored through fast Fourier transform analysis to ensure the noise floor remained within permissible limits throughout the assessment. Audiometric threshold determination strictly followed the modified Hughson–Westlake protocol. Testing commenced at 1,000 Hz, with stimulus intensity reduced in 10 dB steps until no response was detected and increased in 5 dB steps to identify threshold levels. The hearing threshold was defined as the lowest intensity at which participants achieved a response accuracy of no less than 50%. Inconsistent initial thresholds were repeatedly verified to ensure data reliability. Contralateral masking was applied when interaural attenuation exceeded 40 dB. The four-frequency pure-tone average (500, 1,000, 2,000, and 4,000 Hz) was finally calculated to quantitatively evaluate hearing impairment severity. Demographic and clinical characteristics of all participants are summarized in Table 1.

**TABLE 1:** General information and cognitive-related scores of patients.

Base date	Presbycusis (*n* = 129)		HC (*n* = 121)		*p-*value
Age (years)	63.55 ± 3.49		64.10 ± 3.44		0.207
Gender (male:female)	67:62		68:53		0.499
Educational level (years)	11.66 ± 2.18		11.31 ± 1.98		0.180
Hearing (dB HL)(L\R)	32.80 ± 4.14	32.85 ± 6.43	15.48 ± 2.56	16.05 ± 3.05	< 0.001
Hearing average (dB HL)	32.82 ± 3.82		15.76 ± 2.02		< 0.001
Cognitive performance					
MMSE	28.82 ± 0.99		29.01 ± 1.14		0.149
MOCA	26.54 ± 1.97		27.28 ± 1.25		< 0.001
AVLT	34.81 ± 7.81		35.69 ± 6.71		0.344
AVLTd	7.01 ± 2.40		6.79 ± 2.40		0.464
CFT	32.16 ± 6.04		34.45 ± 1.77		< 0.001
CFTd	18.23 ± 4.51		21.37 ± 5.66		< 0.001
DST	11.31 ± 1.82		11.58 ± 1.84		0.262
TMT-A	72.65 ± 26.91		66.05 ± 16.77		0.024
TMT-B	194.39 ± 55.61		177.37 ± 52.05		0.013
CDT	3.55 ± 0.54		3.50 ± 0.55		0.432
VFT	14.30 ± 3.75		14.48 ± 3.30		0.698
DSST	69.42 ± 8.40		69.69 ± 9.01		0.803

### Neuropsychological assessment

A series of neuropsychological tests were performed on all patients to test their cognitive functions. These tests include the Mini-Mental State Examination (Pangman et al., 2000), the Montreal Cognitive Assessment (MoCA) (Ganguli et al., 2010) to evaluate general cognitive function, the Auditory Verbal Learning Test (AVLT) and AVLT-d (Stein et al., 2012) to test episodic language learning, the Complex Figure Test (CFT and CFT-d) for visual memory recall (Peña-Casanova et al., 2009); and the digital span test (DST) for testing language working memory (Leung et al., 2011). In addition to testing psychological processing speed, executive function tests were also performed through Trail-Making Tests A and B (TMT-A and TMT-B) (Perrochon and Kemoun, 2014) and the Clock Drawing Test (CDT) (Park et al., 2018). Visual spatial ability was evaluated through the digital symbol substitution test (DSST) (Daderwal et al., 2022) and the verbal fluency test (VFT) (Sciarma et al., 1990). These tests are based on international standards, a total of 12 items, which take approximately 50 min, and that could not be completed were excluded.

### MRI data acquisition

All participants were fully informed of the scanning procedure, experimental purpose, and safety precautions before MRI acquisition. Foam padding was used to restrict head motion, and earplugs were provided to reduce scanner noise. Participants were instructed to remain awake, keep their eyes closed, maintain a relaxed state, and avoid active thinking during scanning. High-resolution T1-weighted structural images were acquired using a three-dimensional volumetric sequence with the following parameters: TR 12 ms, TE 5.1 ms, inversion time 450 ms, flip angle 15, slice thickness 1 mm, no gaps, FOV 240 mm × 240 mm, matrix size 256 × 256, voxel size 1 mm × 1mm × 1 mm; number of slices 172, and total scan time 5 min and 20s (Shang et al., 2021).

### Data processing and analysis

#### Whole-brain volume data processing

The software used for the research is based on statistical parameter mapping (SPM 12), and the resting state fMRI data analysis toolkit RESTplus V1.25,^1^ which are all based on the Matelab R2022a platform.

#### Data preprocessing

Structural image preprocessing was performed using SPM12 and RESTplus V1.25 toolkit based on the MATLAB R2022a platform. All T1-weighted images were segmented into gray matter, white matter, and cerebrospinal fluid using the unified segmentation model. Gray matter concentration maps were initially affine-registered to the standard MNI space, followed by nonlinear deformation and resampling to a voxel resolution of 1.5 mm × 1.5 mm × 1.5 mm. The GMV of each voxel was obtained by multiplying the GM concentration graph by the nonlinear determinant obtained by the spatial normalization step. The GMV images were subsequently smoothed with a Gaussian kernel of 6 mm × 6 mm × 6 mm FWHM. Finally, after spatial preprocessing of the data, the normalized, modulated and smooth GMV maps were used for statistical analysis (Shang et al., 2021).

### Global network parameters and nodal network metrics

The automated anatomical labeling (AAL) template was employed to parcellate the brain into 90 anatomical regions, each defined as a network node. GMV values for each node were extracted from individual MRI images. To minimize confounding effects, a linear regression model was applied to adjust the GMV values for covariates including age, sex, and years of education. This preprocessing step ensured that subsequent network analyses reflected intrinsic structural covariance patterns rather than demographic variability (Zhu et al., 2024). Subsequently, we obtained the GMV residuals, and define the residuals between nodes as edges of the network. Networks are typically characterized by global network parameters and regional node parameters. These parameters mainly consist of small - world properties and network efficiency parameters. The global properties include the clustering coefficient (Cp), which quantifies the degree to which nodes in a network tend to cluster together, and the characteristic path length (Lp), representing the average shortest path length between all pairs of nodes. We also calculated the normalized clustering coefficient (γ) and normalized characteristic path length (λ) relative to matched random networks to determine small-worldness (σ), a metric indicating a balance between local clustering and global integration. Furthermore, global network efficiency (Eglob) and local network efficiency (Eloc) were computed to evaluate the network’s capacity for parallel information transfer and fault tolerance, respectively. To investigate nodal centrality, we utilized betweenness centrality (Bc) quantifies the extent to which a node lies on the shortest paths between other nodes in the network, serving as a critical bridge or hub for information flow, degree centrality (Dc) measures the number of direct connections (edges) a node possesses, reflecting its immediate local connectivity or topological importance, and node efficiency (Ne) evaluates the capability of a single node to exchange information with all other nodes in the network, calculated as the average of the inverse shortest path lengths from the node to all others (Onias et al., 2014).

Based on a review of prior literature, we extract and define the process through the following three steps. We extract and define the process through the following three steps. These steps include: (1). Atlas Mapping: We mapped the canonical functional coordinates of the five networks to the AAL-90 anatomical labels. Based on prior literature and functional connectivity atlases, specific AAL nodes were assigned to each functional network. For instance, the default mode module network was defined by aggregating nodes including the medial prefrontal cortex, posterior cingulate cortex/precuneus, and bilateral inferior parietal lobules. (2). Sub-matrix extraction: For each subject’s 90*90 connectivity matrix, we extracted the specific sub-matrices corresponding to the indices of the nodes assigned to each of the five networks. This resulted in five distinct sub-networks for each participant. (3). Network Assessment: The topological properties of these sub-networks were evaluated by calculating the nodal metrics-specifically Bc, Dc, and Ne averaged across all constituent nodes within each network. This approach allowed for the quantification of information flow and centrality specifically within the (1) VN: Visual network; (2) SMN: sensorimotor network; (3) DMN: default mode module network; (4) SN: subcortical network; (5) FPN: frontal parietal network, independent of the global whole-brain topology (Huang et al., 2023).

### Statistical analysis

This study adopted a unified and standardized statistical analytical framework for all data analyses. To minimize demographic confounding bias, healthy controls were matched to presbycusis patients at the group level via frequency matching according to age, sex, and years of education, rather than one-to-one individual pairwise matching. All statistical analyses were performed using SPSS 26.0 and customized MATLAB scripts, with detailed analytical strategies specified as follows. First, demographic and behavioral statistical analysis. Independent-samples *t*-tests were applied for continuous demographic and neuropsychological variables, while chi-square tests were utilized for categorical variables. All quantitative data were presented as mean ± standard deviation, and a two-tailed *p*-value less than 0.05 was defined as the threshold for statistical significance. Second, intergroup comparison of network topological properties. Non-parametric permutation tests with 1,000 random iterative permutations were performed to detect group differences in global and nodal topological parameters of structural covariance networks, with the significance level set at α = 0.05. Third, multiple comparison correction. FDR correction with a threshold of *q* < 0.05 was implemented to control type I errors induced by multiple statistical tests across 90 brain nodes. Fourth, null hypothesis network modeling. Random null network models were constructed by retaining the original number of network nodes and edges while randomly rewiring inter-node connections. A total of 1,000 randomized null networks were generated to verify whether the topological properties of the observed brain structural networks significantly deviated from random network organization.

We further validated the effectiveness and reliability of the frequency-matching design by comparing baseline demographic characteristics between the two groups using independent-samples *t*-tests for continuous variables and chi-square tests for categorical variables. Given that sex serves as a well-recognized critical confounding factor in auditory and cognitive neuroscience research, sex was incorporated as a covariate in multivariate regression models to eliminate potential residual confounding effects, despite group-level frequency matching. In contrast, age and educational level were fully balanced between groups after matching procedures, so unadjusted statistical tests were adopted for subsequent intergroup comparisons, which strictly conforms to the standardized analytical specifications for frequency-matched observational clinical studies.

## Results

A total of 129 patients diagnosed with presbycusis were included in the experimental group, whereas 121 HCs without hearing impairment were included in the control group. The presbycusis group had a mean age of 63.55 ± 3.49 years with a male-to-female ratio of 1.08:1, whereas the control group had comparable demographic characteristics, with a mean age of 64.10 ± 3.44 years and a male-to-female ratio of 1.28:1. Consistent with the frequency-matching design, the two groups demonstrated no significant differences in age, sex, or years of education (all *p* > 0.05), thereby validating the efficacy of our recruitment and matching strategy. However, significant differences in hearing thresholds were detected between the groups: the presbycusis group presented a mean hearing threshold of 32.82 ± 3.82 dB HL, which was significantly greater than that of the control group 15.76 ± 2.02 dB HL (*p* < 0.001). The two groups exhibited significant differences in cognitive performance, as evidenced by substantial disparities in MoCA, CFT, CFTd, TMT-A, and TMT-B scores, all of which were statistically significant (*p* < 0.05) (Table 1).

### Results of network topological properties

In small-world network properties: Cp, γ, and Eloc reflect the functional segregation of the network (i.e., local organizational capacity); Lp, λ, and Eglob reflect the functional integration of the network (i.e., global information processing capacity); σ quantifies the balance between Lp and Cp. The results showed that there were no significant overall changes in the global network topological properties of patients with presbycusis, but significant differences were observed in the topological properties of specific nodes (Table 2).

**TABLE 2:** The global and local information processing capacity of the SCN.

Group	Cp	Lp	σ	λ	γ	Eglob	Eloc
HC	0.2596	0.7511	0.4201	0.4501	0.4760	0.2222	0.3064
PA	0.2656	0.7294	0.4353	0.4320	0.4719	0.2281	0.3103
p	0.4190	0.0429	0.5609	0.0411	0.9020	0.4160	0.6400

Normalized clustering coefficient (γ), normalized characteristic path length (λ), small worldness (σ), clustering coefficient (Cp), characteristic path length (Lp), global efficiency (Eglob), and local efficiency (Eloc).

**FIGURE 1:**
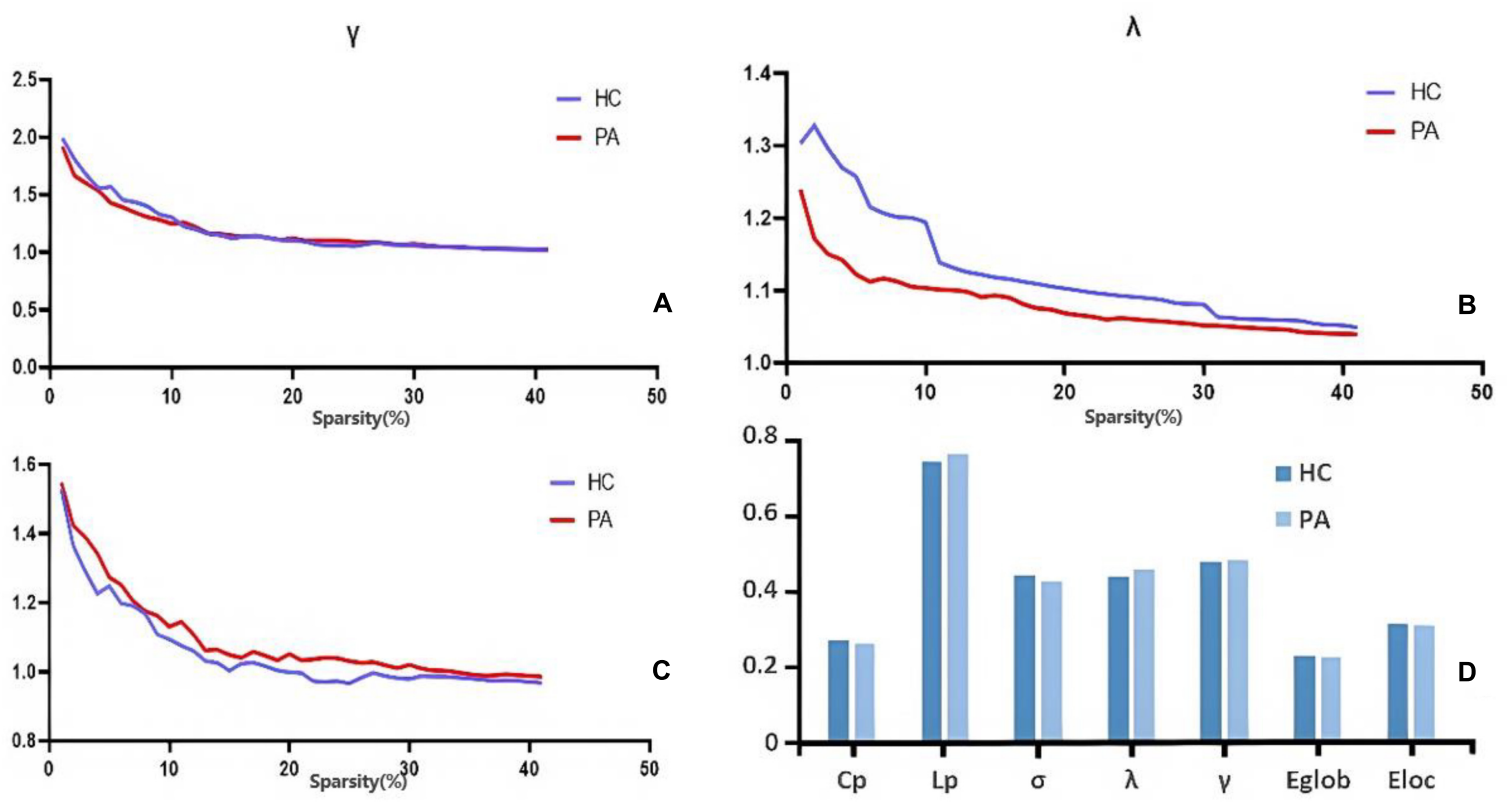
Changes in γ, λ, σ changes between the presbycusis patient group and the healthy control group **(A–C)**. Differences between the two groups of patients in terms of characteristic path length **(D)**.

Significant differences were observed between the two groups in terms of Lp andλ (*p* < 0.05 after FDR correction). Compared with the healthy control (HC) group, patients with presbycusis exhibited a significant decrease in Lp (HC: 0.7511 ± 0.032; PA: 0.7294 ± 0.028; *p* = 0.0429) and a significant decrease in λ (HC: 0.4501 ± 0.021; PA: 0.4320 ± 0.019; *p* = 0.0411). Compared with the matched random networks, λ in the patient group was significantly lower than that in the random network (*p* < 0.01), while there was no significant difference between λ in the control group and the random network. This suggests that the improved efficiency is a specific change associated with presbycusis (Figure 1).

### Results of node topological properties

**FIGURE 2:**
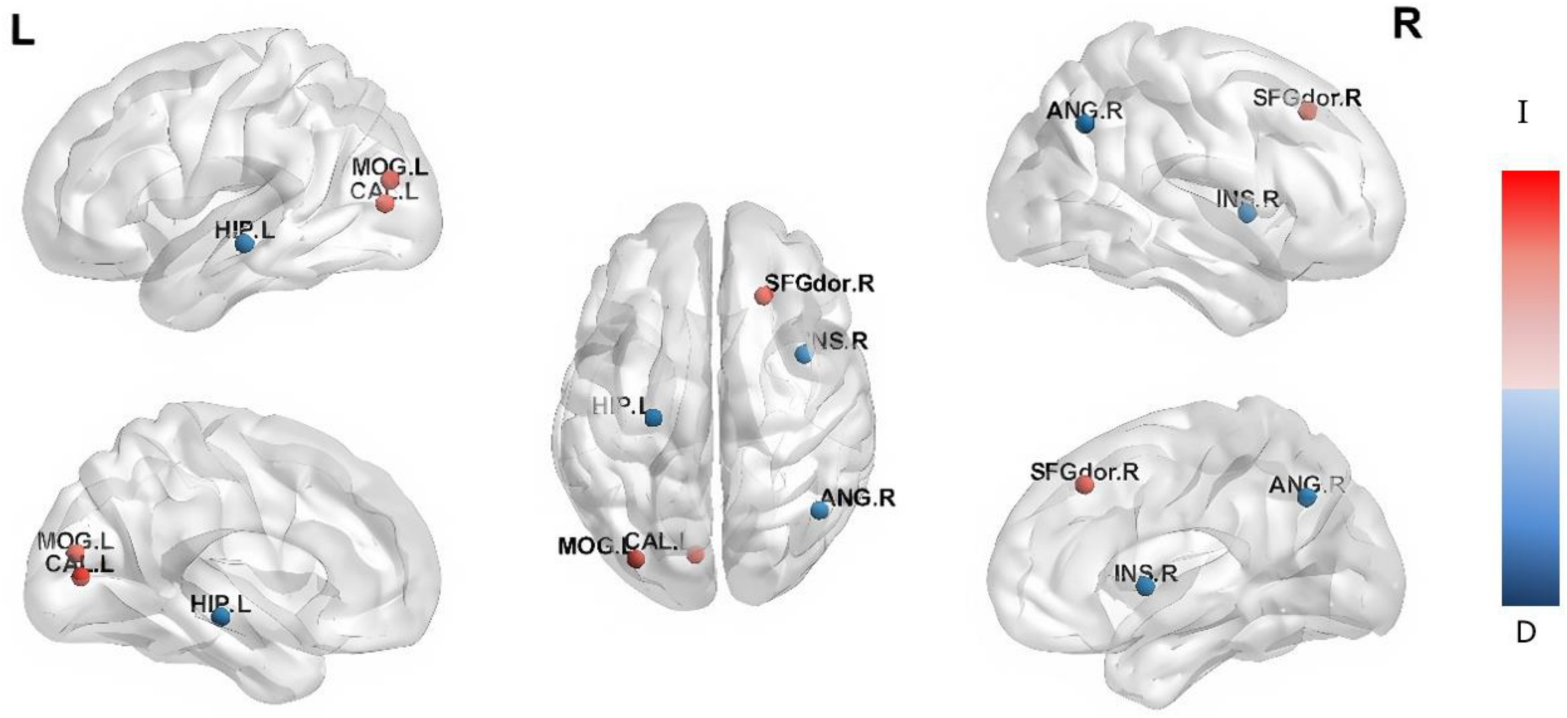
The nodes of the brain regions that have undergone changes, with red representing an increase and blue representing a decrease. The Dc of the bilateral superior frontal gyrus was significantly increased; The Ne of the right insula and left hippocampus was significantly decreased; The Bc of the occipital cortex (including the calcarine sulcus and cuneus) was significantly increased. I, increased; D, decreased.

Alterations in node topological properties were observed in multiple brain regions of patients with presbycusis, including the right insula, left hippocampus, bilateral superior frontal gyrus (SFG), and occipital cortex (Figure 2). Specifically: The Dc of the bilateral superior frontal gyrus was significantly increased (*p* < 0.05, FDR-corrected); The Ne of the right insula and left hippocampus was significantly decreased (*p* < 0.05, FDR-corrected); The Bc of the occipital cortex (including the calcarine sulcus and cuneus) was significantly increased (*p* < 0.05, FDR-corrected). These changes were mainly concentrated in the cognitive control network (SFG), auditory-emotional integration network (insula, hippocampus), and visual processing network (occipital cortex) (Figure 2).

## Discussion

The present work investigated the differences in brain SCNs and cognitive function between presbycusis patients and normal controls. The key findings of our research are as follows: First, comparative analysis revealed significant cognitive decline in presbycusis patients relative to healthy controls, particularly in the executive function and memory domains. Second, while the global network topological properties remained unchanged in presbycusis patients, we observed significant alterations in specific nodal network topological properties. Third, comprehensive analysis of node attributes revealed distinct patterns in presbycusis patients compared with healthy controls.

GMV covariance, the statistical foundation of SCNs, reflects coordinated microstructural changes driven by shared neurobiological processes. At the cellular level, these macroscopic patterns likely arise from axonal demyelination and dendritic pruning, which disrupt the structural integrity and connectivity between cortical regions (Alexander-Bloch et al., 2013). Furthermore, synaptic loss acts as a critical factor, potentially weakening the neurotrophic interactions that maintain volumetric coordination across functionally connected networks (Zuo et al., 2005). Under chronic auditory sensory deprivation, such microstructural deteriorations may also trigger retrograde neuronal degeneration, wherein insufficient afferent auditory input induces secondary morphological atrophy in downstream associative cortical regions (Wang et al., 2016). Accordingly, disrupted SCN covariance patterns observed in presbycusis can be regarded as non-invasive in vivo neuroimaging biomarkers that mirror underlying histopathological alterations, representing disrupted coordination in the development, maintenance, and stabilization of large-scale neural circuits.

Building upon these mechanistic insights, the SCNs alterations identified in presbycusis likely represent a chronic, large-scale adaptation to progressive sensory deprivation, distinct from patterns observed in other etiologies of hearing loss. Unlike the acute changes characteristic of noise-induced hearing loss (NIHL) or the profound developmental reorganization seen in congenital deafness, presbycusis involves a slow, age-related decline that triggers a unique compensatory response. For instance, recent diffusion tensor imaging (DTI) studies on NIHL have revealed significant microstructural degradation in specific association fibers, such as the cingulum and the left inferior longitudinal fasciculus, while notably sparing the auditory cortex and arcuate fasciculus (Zolkefley et al., 2024). This suggests that NIHL exerts a differential impact on white matter tracts related to cognitive and emotional processing rather than causing diffuse structural damage. In contrast, the observed increase in visual network centrality in our presbycusis cohort aligns more closely with functional MRI evidence of cross-modal plasticity, wherein visual regions are recruited to compensate for deteriorating auditory function (Peelle et al., 2011; Chen et al., 2014). Such large-scale structural reorganization is presumably mediated by integrated white matter fiber pathways; future multimodal imaging studies combining DTI and SCN analyses are warranted to verify microstructural tract integrity underlying these covariance alterations and further clarify the compensatory neural mechanisms. Collectively, the present findings demonstrate that SCN abnormalities in presbycusis constitute a unique age-related neuroanatomical signature, which is fundamentally differentiated from the tract-specific vulnerability pattern observed in noise-induced hearing impairment.

Consistent with previous clinical evidence, presbycusis patients exhibited significant cognitive impairments relative to healthy controls, particularly in visual retention and reproduction abilities assessed by the Complex Figure Test (CFT/CFT-d) and complex cognitive flexibility and task-switching performance measured by the Trail-Making Test B (TMT-B), reflecting impairments in visual-spatial reasoning and executive function. Notably, while MMSE scores did not differ between groups, MoCA scores were significantly lower in patients, which may be attributed to the higher sensitivity of MoCA in detecting subtle executive and visuospatial decline (Ganguli et al., 2010). These cognitive alterations may be linked to disrupted audio-visual integration due to hearing loss, which compromises synergistic sensory processing and reduces efficiency of visual memory encoding (Li et al., 2015). Such functional impairments align with reported disruptions in functional network connectivity and neurochemical reorganization in presbycusis (Xing et al., 2020; Li et al., 2023), and further underscore the importance of investigating concomitant structural covariance network changes to fully elucidate the neural mechanisms underlying this impairment-compensation dynamic.

From the perspective of graph theory, human brain neural interactions are organized as complex small-world networks that balance local specialization and global integration, rather than purely regular or random networks (Shang et al., 2023) Graph theory analysis revealed significantly lower characteristic Lp and λ in the presbycusis group compared to healthy controls. Since Lp is inversely related to global communication efficiency (Rubinov and Sporns, 2010), its reduction indicates shortened information transmission pathways and enhanced global network integration. Crucially, the patient group’s λ was significantly lower than that of matched random networks, whereas the control group’s λ was not, confirming this efficiency increase as a disease-specific alteration rather than a random fluctuation. This reduction in λ, which quantifies a network’s efficiency relative to a random topology (Watts and Strogatz, 1998; Xing et al., 2020), likely reflects a neuroadaptive, compensatory reorganization of the structural covariance network in response to chronic auditory deprivation. Such topological optimization may facilitate the establishment of more direct connections between auditory-related regions and distributed cortical areas involved in language, memory, and emotional processing. This enhanced integration could support more efficient cross-modal information transfer, potentially aiding comprehension and memory retrieval by accelerating the access to semantic knowledge and visual cues to compensate for degraded auditory input (Kokudai et al., 2021). These structural topological alterations are highly consistent with previously reported functional network reorganization in presbycusis, highlighting the essential role of large-scale structural network plasticity in mediating brain compensatory mechanisms.

Widespread nodal topological disruptions were detected across multiple functional modules, which collectively contributed to the adaptive global topological alterations observed in presbycusis patients. Significant intergroup differences in nodal topological organization were identified across five core functional subnetworks, including the FPN, DMN, SMN, VN, and SN. These findings are highly congruent with previous research reported by Huang et al., which documented abnormal network alterations in the DMN, subcortical network, cingulate-insular network, occipital network, and FPN in age-related hearing loss (Huang et al., 2023). Such cross-study consistency strongly validates the robustness of network-level neural remodeling in presbycusis and further indicates that age-related auditory impairment not only affects low-order sensory processing networks (VN and SN) but also induces extensive adaptive or maladaptive alterations in high-order cognitive regulatory networks (FPN and DMN).

The present study identified extensive modular and nodal topological abnormalities in the structural covariance networks of presbycusis patients. Notably, multiple core brain nodes exhibited aberrant topological properties that subserve the network-level reorganization. Within the cognitive control network, increased degree centrality (Dc) was observed in the bilateral superior frontal gyrus (SFG), a core region responsible for executive control and cognitive resource allocation (Spreng and Turner, 2013). This may signify heightened recruitment of cognitive control resources to support the increased demands of auditory processing under degraded acoustic conditions. Furthermore, the auditory-emotional integration network exhibited decreased Ne in the right insula—a region implicated in auditory-emotional integration—and the left hippocampus, which is crucial for memory encoding (Yun et al., 2020; Pangman et al., 2000). This reduction suggests a deficit in the integration of auditory information and its subsequent consolidation, potentially attributable to compromised audiovisual integrative mechanisms. Regarding the visual processing network, increased Bc in the occipital cortex implies its augmented role as an intermediary hub. This finding supports the cross-modal plasticity hypothesis, wherein visual cortical regions undergo functional adaptation to compensate for auditory deprivation (Ganguli et al., 2010). Such reorganization may account for the relative preservation of elementary visuospatial processing despite coexisting impairments in visual memory. Together, these findings point to widespread and network-specific disruptions in brain morphology, providing further evidence for the network-level pathophysiology underlying the condition.

Several methodological limitations of the present study should be acknowledged. First, the cross-sectional design precludes causal inferences. Longitudinal studies are needed to track SCN dynamics alongside hearing and cognitive changes. Second, we did not stratify patients by hearing loss severity or cognitive status, which may reveal different reorganization patterns. Future studies with larger subsamples should explore these dimensions. Third, nevertheless, it must be acknowledged that residual confounding due to unmeasured covariates remains a potential limitation of this study. Fourth, the covariance network cannot extract individual difference values for correlation analysis. Finally, integrating multimodal imaging (e.g., DTI, fMRI) with SCN analysis would provide a more comprehensive view of structure-function relationships in presbycusis. Subsequently, we can achieve clinical correlation analysis through the algorithm of individual networks.

In conclusion, the results of this study indicate that significant changes occur in gray matter volume (GMV) and structural covariance networks (SCN) across multiple brain regions associated with presbycusis, and these alterations regulate the cognitive, emotional, and sensory functions of patients with presbycusis. Chronic hearing loss may trigger changes in brain volume and neural network remodeling, accompanied by compensatory responses in other sensory systems (e.g., the visual system). Through a rigorous statistical analysis framework, strict control of confounding factors, and support from classic literature, this study confirms the core role of SCN reorganization in presbycusis. These findings provide new evidence for understanding the neural mechanisms of the disease, emphasize the importance of early intervention in protecting patients’ cognitive function, and point out key directions for future research.

## Data Availability

The raw data supporting the conclusions of this article will be made available by the authors, without undue reservation.

## Footnotes

1
http://restfmri.net/forum/index.php
